# Epidemiology and mortality in patients hospitalized for burns in Catalonia, Spain

**DOI:** 10.1038/s41598-023-40198-2

**Published:** 2023-09-01

**Authors:** L. Abarca, P. Guilabert, N. Martin, G. Usúa, Juan P. Barret, Maria J. Colomina

**Affiliations:** 1 Anesthesia and Critical Care Department, Hospital Universitari Vall d’Hebron, 08035 Barcelona, Spain; 2 Anesthesia and Critical Care Department, Hospital Universitari Alicante, Alicante, Spain; 3 Anesthesia and Critical Care Department, Hospital Clinic, Barcelona, Spain; 4 Plastic Surgery Department and Burn Centre, Hospital Universitari Vall d’Hebron, Barcelona, Spain; 5 Department of Anesthesia, Critical Care and Pain Clinic, Hospital Universitari de Bellvitge, Barcelona, Spain

**Keywords:** Epidemiology, Risk factors

## Abstract

Burn injuries are one of the leading causes of morbidity worldwide. Although the overall incidence of burns and burn-related mortality is declining, these factors have not been analysed in our population for 25 years. The aim of this study has been to determine whether the epidemiological profile of patients hospitalized for burns has changed over the past 25 years. We performed a retrospective cohort study of patients hospitalised between 1 January 2011 and 31 December 2018 with a primary diagnosis of burns. The incidence of burns in our setting was 3.68/10^5^ population. Most patients admitted for burns were men (61%), aged between 35 and 45 years (16.8%), followed by children aged between 0 and 4 years (12.4%). Scalding was the most prevalent mechanism of injury, and the region most frequently affected was the hands. The mean burned total body surface (TBSA) area was 8.3%, and the proportion of severely burned patients was 9.7%. Obesity was the most prevalent comorbidity (39.5%). The median length of stay was 1.8 days. The most frequent in-hospital complications were sepsis (16.6%), acute kidney injury (7.9%), and cardiovascular complications (5.9%). Risk factors for mortality were advanced age, high abbreviated burn severity index score, smoke inhalation, existing cardiovascular disease full-thickness burn, and high percentage of burned TBSA. Overall mortality was 4.3%. Multi-organ failure was the most frequent cause of death, with an incidence of 49.5%. The population has aged over the 25 years since the previous study, and the number of comorbidities has increased. The incidence and severity of burns, and the percentage of burned TBSA have all decreased, with scalding being the most prevalent mechanism of injury. The clinical presentation and evolution of burns differs between children and adults. Risk factors for mortality were advanced age, smoke inhalation, existing cardiovascular disease, full-thickness burn, and high percentage of burned TBSA.

## Introduction

Burns are a common problem in people of all age groups, from all walks of life, all over the world.

Nonfatal burns, the most prevalent type, are one of the leading causes of morbidity, including prolonged hospitalization, disfigurement, and disability.

Recent years have seen a significant decrease in both burn incidence and burn-related mortality, although figures continue to be very high. In 2017, burns accounted for 9 million injuries and 120,000 deaths worldwide^1^.

In addition to the decrease in incidence and mortality, a systematic review published by Smolle^2^ reported a downward trend in burn-related length of hospital stay (LOS) between 2001 and 2016. The length of stay/percentage of burned total body surface area (LOS/%TBSA) ratio has decreased from 1.5–3 days to 0.5–1.4 days^2–4^.

A multitude of epidemiological studies published in Europe over the past 10 years have reported a decrease in incidence, mortality, and severity of burns (a major burn is defined as > 20% TBSA^5^) and LOS^6–16^. However, few studies describe comorbidities in relation to burned patients and their effect on morbidity and mortality^17–20^.

The last epidemiological study performed in Catalonia was published in 1999^21^, and showed that comorbidity and female sex were not associated with higher mortality.

*The healthcare system in Catalonia and in Spain is public system.* The Hospital Universitario Vall d'Hebron (HUVH) is a tertiary hospital and there *is only this burn unit that receives patients not only from the local Barcelona area but also from other provinces*, with a catchment area of 9 million inhabitants. It is also one of the leading burn units in Spain. The Hospital has been awarded the CSUR certificate of clinical quality in Spain, is a member of the European network of burns disasters, and is certified by the EBA (European Burns Association).

The aim of this study has been to describe the epidemiological profile of the hospitalised burned patient, the complications occurring during their hospital stay, and the burn-related mortality rate, and to compare these variables with data published 25 years ago.

## Material and method

### Study design and patient selection

The study was approved by the Hospital Universitario Vall d’ Hebron ethics committee (PR[ATR]385/2016). The requirement for informed consent from the study subjects was waived by the IRB of Hospital Universitario Vall d’ Hebron due to the retrospective study. The study follows the recommendations of the STROBE statement for reporting observational studies and all research was performed in accordance with the Declaration of Helsinki.

This is a retrospective cohort study of patients admitted to the HUVH from 1 January 2011 to 31 December 2018.

Inclusion criteria were: all patients admitted to the HUVH with a primary diagnosis of burns. The primary diagnosis was coded according to the criteria of the International Classification of Diseases (the ICD-9 was used from 2011 to 2016 and the ICD-10 from 2017 to 2018).

Patients diagnosed with burns who died within the first 12 h of admission were excluded.

Data were taken from the patient’s digital clinical history and supplemented with data from their primary care records. The cost was calculated using data from the annual budget of the burn unit. Patients were monitored at 30 and 90 days after hospital discharge by the follow-up specialist.

Study variables were (see Appendix 1): Age, sex, burned TBSA, burn location, burn degree, mechanism of injury, work accident, Abbreviated Burn Score Index (ABSI), and smoke inhalation. Comorbidities were: toxic habits, cardiovascular or respiratory disease, diabetes mellitus, chronic kidney failure, liver disease, clotting disorder, use of antiplatelet agents or oral anticoagulants, chronic anaemia, neuromuscular disorders, active neoplasia, and obesity. Variables collected during hospitalisation were: LOS, ICULOS, surgical intervention and reintervention. Complications during admission included compartment syndrome, cardiovascular complications, acute respiratory distress syndrome, need for tracheostomy and time from admission to tracheostomy, days on mechanical ventilation, acute kidney injury (AKI), stroke, and sepsis. Outcomes variables were: In-hospital mortality 12 h after admission, cause of death, and 30- and 90-day mortality.

Smoke inhalation was defined either by clinical suspicion or diagnosed through fibro-bronchoscopy. MOF (Multiple Organs Failure) was defined as the failure of two or more of the following organs or systems: cardiovascular, respiratory, neurological, renal, hematological, gastrointestinal, hepatic, and neurological. We followed the recommendations of the ABA (American Burn Association) for definition of sepsis^22^. AKI (Acute Kidney Injury) diagnosis was based on the RIFLE clinical criteria^23^.The diagnosis of ARDS (Acute Respiratory Distress Syndrome) adhered to the diagnostic criteria outlined in the Berlin definition^24^.

Data were extracted from the Redcap project database (Tennessee, USA).

### Statistical analysis

The Redcap project (https://www.project-redcap.org/) database was used for this study. Statistical analysis was performed on R (version 4.1.1, R Core Team, Vienna, Austria) by the Statistics and Bioinformatics Unit (UEB) of the Vall d'Hebrón Hospital Research Institute.

For categorical variables, the frequency and 95% confidence interval were calculated. For continuous variables, the mean and standard deviation were calculated together with the mean 95% confidence interval and the median and interquartile range (IQR).

A group comparison test was performed to compare the different categories of the variable of interest. The Kruskall–Wallis test was used for quantitative variables. The chi-square test or Fisher's exact test was used for categorical variables when the expected count was less than 5. Kaplan–Meier curves were used to analyse the factors associated with an increase in mortality. A univariate Cox regression model was fitted to quantitative variables. The Hazard Ratio was calculated with a 95% confidence interval. Variables were selected attending at clinical reasons from those related at the univariate analysis in order to avoid overfitting”. For variable selection in automatic model, Lasso technique has been used. This technique penalized the función of maximization of the coeficients. This penalization is lead by a lamda parameter that is maximized to obtain the optimal value and select all the variables over the lambda value.

### Ethics approval and consent to participate

This study was approved by the HUVH ethics committee (PR(ATR)385/2016). The recommendations of the STROBE statement for reporting observational studies were followed.

## Results

**Figure 1:**
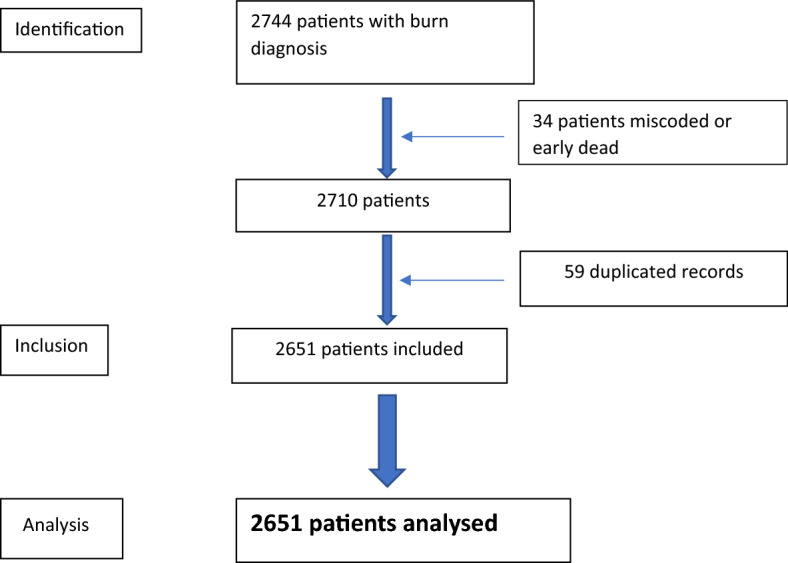
Study flow chart.

A total of 2744 patients were included in the study (Fig. 1). Of these, 34 were misdiagnosed or patients died within 12 h of admission, and therefore eliminated. Two separate admissions (discharged and re-admitted for surgery or burn-related complications) were recorded for 59 patients. These double admissions with their corresponding hospital stays were merged into a single variable. The date of the first admission was taken as the date of admission to simplify the calculation of other time-related variables, such as the time from admission to various procedures.

### Demographics

The incidence of hospital admission was 3.68/100,000 inhabitants (Table 1). Of the 2651 study patients, 2169 were adults (81.8%) and 482 were aged under 16 years (children). Male patients accounted for 61.9% of admissions. Mean age was 41.4 ± 24.7 years. The most numerous age group was 35–45 years in adults (16.8%) and 0–4 years (12.4%) in children.

**Table 1:** Demographic and burn-related variables.

		< 16 year	> 16 year	ALL	*p* value	n	Missing
**Demographic variables**							
		482 (18.18%)	2169 (81.82%)	**2651 (100%)**		2651	0
Age_2_		482 4.1 (4.2) CI (3.8; 4.5) 2.2 (1.3, 5.4)	2169 49.7 (19) CI(48.9; 50.5) 47.4 (34.9, 63.1)	**2651** **41.4 (24.7)** **CI (40.5; 42.4)** **41.5 (24.6,59)**	< 0.001	2651	0
Sex_1_	Men	291 (60.4%) CI (55.9; 64.8)	1343 (61.9%) CI) 59.8; 64)	**1634 (61.6%)** **(59.8; 63.5)**	0.563^3^	2651	0
	Women	191 (39.6%) CI (35.2; 44.1)	826 (38.1%) CI (36; 40.2)	**1017 (38.4%)** **(36.5; 40.2)**			
**Burn-related variables**							
Mechanism_1_	Flame	52 (10.8%) (8.2; 13.9)	880 (40.6%) (38.5; 42.7)	**932 (35.2%)** **(33.3; 37)**	< 0.001	2651	0
	Scalding	369 (76.6%) (72.5; 80.3)	630 (29%) (27.1; 31)	**999 (37.7%)** **(35.8; 39.6)**			
	Explosion	5 (1%) (0.3; 2.4)	150 (6.9%) (5.9; 8.1)	**155 (5.8%)** **(5; 6.8)**			
	Electrocution	23 (4.8%) (3; 7.1)	151 (7%) (5.9; 8.1)	**174 (6.6%)** **(5.7; 7.6)**			
	Contact	25 (5.2%) (3.4; 7.6)	146 (6.7%) \ (5.7; 7.9)	**171 (6.5%)** **(5.5; 7.5)**			
	Chemicals	3 (0.6%) (0.1; 1.8)	166 (7.7%) (6.6; 8.9)	**169 (6.4%)** ** (5.5; 7.4)**			
	Unknown	2 (0.4%) (0.1; 1.5)	17 (0.8%) (0.5; 1.3)	**19 (0.7%)** **(0.4; 1.1)**			
	Other	3 (0.6%) (0.1; 1.8)	29 (1.3%) (0.9; 1.9)	**32 (1.2%) ** **(0.8; 1.7)**			
Location_1_						2651	0
	Head and neck_1_	204 (42.3%) (37.9; 46.9)	732 (33.7%) (31.8; 35.8)	**936 (35.3%) ** **(33.5; 37.2)**	< 0.001		
	Arms_1_	297 (61.6%) (57.1; 66)	1421 (65.5%) (63.5; 67.5)	**1718 (64.8%) ** **(63; 66.6)**	0.117		
	Chest	187 (38.8%) (34.4; 43.3)	485 (22.4%) (20.6; 24.2)	**672 (25.3%) ** **(23.7; 27.1)**	< 0.001		
	Abdomen	71 (14.7%) (11.7; 18.2)	373 (17.2%) (15.6; 18.9)	**444 (16.7%) ** **(15.3; 18.2)**	0.213		
	Back	62 (12.9%) (10; 16.2)	274 (12.6%) (11.3; 14.1)	**336 (12.7%) ** **(11.4; 14)**	0.951		
	Legs	187 (38.8%) (34.4; 43.3)	1187 (54.7%) (52.6; 56.8)	**1374 (51.8%) ** **(49.9; 53.7)**	< 0.001		
	Genitals_1_	29 (6%) (4.1; 8.5)	77 (3.6%) (2.8; 4.4)	**106 (4%) ** **(3.3; 4.8)**	0.018		
**Burn-related variables**							
Season	Winter	123 (25.5%) CI (21.7; 29.7)	503 (23.2%) CI (21.4; 25)	**626 (23.6%)** **(22; 25.3)**	0.379^3^	2651	0
	Spring	132 (27.4%) CI (23.5; 31.6)	561 (25.9%) CI (24; 27.8)	**693 (26.1%)** **(24.5; 27.9)**			
	Summer	132 (27.4%) CI (23.5; 31.6)	609 (28.1%) CI (26.2; 30)	**741 (28%)** **(26.2; 29.7)**			
	Autumn	95 (19.7%) CI (16.2; 23.5)	496 (22.9%) CI (21.1; 24.7)	**591 (22.3%)** **(20.7; 23.9)**			
Transfer from another centre_1_	Yes	265 (55.2%) (50.6; 59.7)	950 (43.9%) (41.8; 46)	**1215 (46%) ** **(44; 47.9)**	< 0.001	2644	7
Delay_1_	< 6 h	336 (70.7%) (66.4; 74.8)	1149 (54.1%) (51.9; 56.2)	**1485 (57.1%) ** **(55.2; 59.1)**	< 0.001	2599	52
	6 h to 12 h	37 (7.8%) (5.5; 10.6)	186 (8.8%) (7.6; 10)	**223 (8.6%) ** **(7.5; 9.7)**			
	12 h to 24 h	15 (3.2%) (1.8; 5.2)	95 (4.5%) (3.6; 5.4)	**110 (4.2%) ** **(3.5; 5.1)**			
	> 24 h	87 (18.3%) (14.9; 22.1)	694 (32.7%) (30.7; 34.7)	**781 (30.1%) ** **(28.3; 31.9)**			
Work-related burn_1_	Yes	0 (0%) (0; 1.5)	**286 (14.1%)** **(12.6; 15.7)**	**286 (12.5%) ** **(11.2; 14)**	< 0.001	2651	0
%TBSA_2_		6.9 (7.4) (6.2; 7.6) 5 (2.5, 8.4)	8.6 (12.8) (8; 9.1) 4.5 (1.5, 10)	**8.3 (12.1) ** **(7.8; 8.7)** **5 (2, 9.5)**	0.128	2651	0
TBSA_1_	0–10%	372 (77.2%) (73.2; 80.9)	1620 (74.7%) (72.8; 76.5)	**1992 (75.1%)** **(73.5; 76.8)**	< 0.001	2651	0
	> 20%	26 (5.4%) CI (3.6; 7.8)	230 (10.6%) CI (9.3; 12)	**256 (9.7%)** **(8.6; 10.8)**	< 0.001	2651	0
Smoke inhalation_1_	Yes	4 (0.8%) (0.2; 2.2)	139 (6.5%) (5.5; 7.6)	**143 (5.5%) ** **(4.6; 6.4)**	< 0.001	2610	41
Full-thickness burn_1_	Yes	35 (7.3%) CI (5.1; 10)	507 (23.4%) CI (21.6; 25.2)	**542 (20.4%)** **(18.9; 22)**	< 0.001	2651	0
ABSI_2_		2.8 (1) (2.7; 2.9) 3 (2, 3)	5 (1.9) (5; 5.1) 5 (4, 6)	**4.6 (1.9) ** **(4.6; 4.7)** **4 (3.6)**	< 0.001	2648	3

1: n(%)(Exact CI )* p* value: ^4^Chi-2: N mean(sd)(mean 95% CI)median(IQR) * p* value: ^3^Chi-squared.

Significant values are in bold.

### Burn characteristics

The mean burned TBSA was 8.3% (Table 1) full-thickness burns were diagnosed in 23.4% of adults and 7.3% of children. In 75.1% of patients, less than 10% of TBSA was affected. In 9.7% of patients, > 20% of TBSA was affected; in adults, this incidence (10.6%) was double that of children (*p* < 0.001). In all age group, burns in the arms (64.8%) were most frequent, followed by the legs (51.8%), and the head and neck (35.3%).

Scalding (37.7%) was the most prevalent mechanism of injury overall; however, after adjusting for age and sex, the most prevalent mechanism of injury in adult males was flame (39%), and scalding in adult females and in children (45.9% and76.6%, respectively); 14.1% of burns occurred in the workplace. Summer was the season with the highest number of admissions (28%).

### Comorbidities

In adults, 31.5% of patients were smokers and 8.4% reported alcohol dependence. Obesity was the most prevalent comorbidity (39.5%), along with cardiovascular disease (29.9%) and dyslipidaemia (21.1%). In children, respiratory disease (asthma) was the most prevalent comorbidity (7.3%) (Table 2).

**Table 2:** Comorbidities.

Comorbidities	< 16 year	> 16 year	ALL	n	Missing
Cardiovascular_1_	4 (0.8%) CI (0.2; 2.1)	649 (29.9%) CI (28; 31.9)	**653 (24.7%)** **(23; 26.3)**	2649	2
Respiratory_1_	35 (7.3%) CI (5.1; 10)	260 (12%) CI (10.7; 13.5)	**295 (11.2%)** **(10; 12.4)**	2644	2
Diabetes mellitus_1_	2 (0.4%) CI (0.1; 1.5)	260 (12%) CI (10.7; 13.4)	**262 (9.9%)** **(8.8; 11.1)**	2649	2
Chronic kidney injury_1_	0 (0%) CI (0; 0.8)	85 (3.9%) CI (3.1; 4.8)	**85 (3.2%)** **(2.6; 4)**	2649	2
Liver disease_1_	0 (0%) CI (0; 0.8)	57 (2.6%) CI (2; 3.4)	**57 (2.2%)** **(1.6; 2.8)**	2649	2
Clotting disorders/OAC’s/APT_1_	1 (0.2%) CI (0; 1.2)	204 (9.4%) CI (8.2; 10.7)	**205 (7.7%)** **(6.7; 8.8)**	2649	2
Active neoplasm_1_	0 (0%) CI (0; 0.8)	42 (1.9%) CI (1.4; 2.6)	**42 (1.6%)** **(1.1; 2.1)**	2649	2
Neuromuscular disorders_1_	7 (1.5%) CI (0.6; 3)	58 (2.7%) CI (2; 3.4)	**65 (2.5%)** **(1.9; 3.1)**	2649	2
Obesity_1_	10 (3.9%) CI (1.9; 7.1)	617 (39.5%) CI (37.1; 42)	**627 (34.5%)** **(32.3; 36.8)**	1816	835
Chronic anaemia_1_	1 (0.2%) CI (0; 1.2)	77 (3.6%) CI (2.8; 4.4)	**78 (2.9%)** **(2.3; 3.7)**	2649	2
Dyslipidaemia_1_	1 (0.2%) CI (0; 1.2)	456 (21.1%) CI (19.4; 22.8)	**457 (17.3%)** **(15.8; 18.8)**	2647	4
Toxic habits					
Smoking_1_	1 (0.2%) CI (0; 1.2)	684 (31.5%) CI (29.6; 33.5)	**685 (25.8%)** **(24.2; 27.6**	2651	0
Cannabis_1_	0 (0%) CI (0; 0.8)	95 (4.4%) CI (3.6; 5.3)	**95 (3.6%)** **(2.9; 4.4)**	2651	0
Enolism_1_	1 (0.2%) CI (0; 1.2) 184	183 (8.4%) CI (7.3; 9.7)	**184 (6.9%)** **(6; 8)**	2651	0
Cocaine_1_	0 (0%) CI (0; 0.8)	59 (2.7%) CI (2.1; 3.5)	**59 (2.2%)** **(1.7; 2.9)**	2651	0
Other	0 (0%) CI (0; 0.8)	34 (1.6%) CI (1.1; 2.2)	**34 (1.3%)** **(0.9; 1.8)**	2651	0

1: n(%)(Exact CI)* p* value: ^4^Chi-2: N mean(sd) (mean 95% CI)median(IQR) *p *value: Mann–Whitney U.

*OAC’s* Oral anticoagulants, *APT* Antiplatelet therapy.

Significant values are in bold.

### Treatment

Table 3 summarizes the treatments administered. Over half (63.8%) of the sample underwent surgery; the difference between children (41.4%) and adults (68.9%) was statistically significant (*p* < 0.001). Of the surgical patients, 19.5% required a median of 1 (IQR 1.2) reintervention. Median time from the burn event to surgery was 16 [IQR 12–21.8] days in children and 14 [IQR 9–20] days in adults (*p* < 0.001). In contrast, Table 4 shows that the median waiting time for surgery is 10 days [IQR 7–15] days, compared to patients with minor burns, with a median of 15 days (*p* < 0.001).

**Table 3:** Treatment.

Treatment	< 16 year	> 16 year	ALL	*p* value	n	Missing
Surgery_1_	198 (41.4%) (37; 46)	1450 (68.9%) (66.8; 70.8)	**1648 (63.8%)** **(61.9; 65.6)**	< 0.001	2584	67
Reintervention_1_	31 (16.3%) (11.4; 22.4)	283 (19.9%) (17.9; 22.1)	**314 (19.5%)** **(17.6; 21.5)**	0.283^4^	1612	36
Total number of Reintervention_2_	31 1.9 (1.8) (1.3; 2.6) 1 (1, 2.5)	283 1.9 (1.5) (1.7; 2) 1 (1, 2)	**314** **1.9 (1.5)** **(1.7; 2**) **1 (1, 2)**	0.663	314	0
Time from burn to Surgery_2_	198 20.2 (25.6) CI (16.6; 23.8) 16 (12, 21.8)	1449 18.3 (30.5) CI (16.8; 19.9) 14 (9, 20)	**1647** **18.6 (29.9 CI )** **(17.1; 20)** **14 (10, 20)**	< 0.001	1647	1

1: n(%)(Exact CI)* p* value: ^4^Chi-2: N mean(sd) (mean 95% CI)median(IQR) *p* value: Mann–Whitney U.

**Table 4:** Median Days burn until surgery by TBSA—Kruskall–Wallis Test.

TBSA	Days to surgery (Median)	IQR	*p* value
[0,10)	15	[10, 22]	< 0.001
[10,20)	12	[9, 17]	
[20,99]	10	[7, 15]	

### Complications/Outcomes

Some (13%) patients required admission to the ICU; this percentage was significantly higher in children (25.9%) than in adults (10.7%) (*p* < 0.001), although children remained in the unit for a median of 1 (IQR 1.4) day and adults for a median of 13 [IQR 3.30] days (Table 5).

**Table 5:** Complications and outcomes.

		< 16 year	> 16 year	ALL	*p* value	n	Missing
Complications							
LOS ICU_2_		125 5.8 (13.5) CI (3.4; 8.2) 1 (1, 4)	233 20.7 (25.5) CI (17.4; 24) 13 (3, 30)	**358** **15.5 (23.2)** **CI (13.1; 17.9)** **5 (1.21. 8)**	< 0.001	358	0
Days on mechanical ventilation_2_		19 13 (18.7) CI (4; 22) 6 (3.5. 15.5)	204 16.9 (19.7) CI(14.2; 19.6) 10 (2, 25)	**223** **16.6 (19.6 CI)** **(14; 19.2)** **10 (2, 24)**	0.379	223	0
Compartmental syndrome_1_	Yes	0 (0%) CI (0; 0.8)	77 (3.6%) CI (2.9; 4.5)	**77 (2.9%)** **(2.3; 3.7)**	< 0.001	2612	39
Abdominal compartment syndrome_1_	Yes	0 (0%) CI (0; 0.8)	26 (1.2%) CI (0.8; 1.8)	**26 (1%)** **(0.7; 1.5)**	0.009^3^	2607	44
Cardiovascular_1_	Yes	1 (0.2%) CI (0; 1.2)	125 (5.9%) CI (4.9; 7)	**126 (4.8%)** **(4; 5.7)**	< 0.001	2611	40
ARDS_1_	Yes	4 (0.8%) CI (0.2; 2.1)	85 (4%) CI (3.2; 4.9)	**89 (3.4%)** **(2.7; 4.2)**	< 0.001	2613	38
Tracheostomy_1_	Yes	3 (0.6%) CI (0.1; 1.8)	97 (4.6%) CI (3.7; 5.5)	**100 (3.8%)** **(3.1; 4.6)**	< 0.001	2612	39
Days since admission to tracheostomy_2_		3 13 (1.7) CI (8.7; 17.3) 14 (12.5, 14)	94 7.6 (4.9) CI (6.6; 8.6) 7 (4, 11)	**97** **7.8 (4.9 CI)** **(6.8; 8.8)** **7 (4, 11)**	0.035	97	3
Acute kidney injury_1_	Yes	0 (0%) CI (0; 0.8)	167 (7.9%) CI (6.7; 9.1)	**167 (6.4%)** **(5.5; 7.4)**	< 0.001	2606	45
Sepsis_1_	Yes	72 (14.9%) CI (11.9; 18.4)	354 (16.6%) CI (15.1; 18.3)	**426 (16.3%)** **(14.9; 17.8)**	0.404^4^	2612	39
Stroke_1_	Yes	0 (0%) CI (0; 0.8)	14 (0.7%) CI (0.4; 1.1)	**14 (0.5%)** **(0.3; 0.9)**	0.087^3^	2614	37
Blood transfusion_1_	Yes	38 (7.9%) CI (5.7; 10.7)	322 (15%) CI (13.5; 16.6)	**360 (13.7%)** **(12.4; 15.1)**	< 0.001	2627	24
Thrombosis	Yes	1 (0.2%) CI (0; 1.2)	15 (0.7%) CI (0.4; 1.2)	**16 (0.6%)** **(0.4; 1)**	0.333	2612	39
DVT/PE_1_	DVT	1 (100%) CI (2.5; 100)	10 (83.3%) CI (51.6; 97.9)	**11 (84.6%)** **(54.6; 98.1)**	1^3^	13	3
	PE	0 (0%) CI (0; 97.5)	2 (16.7%) CI (2.1; 48.4)	**2 (15.4%)** **(1.9; 45.4)**			
Outcomes							
LOS_2_		482 11.8 (14.8) (10.5; 13.1) 8 (3, 17)	2169 15.2 (21.4) (14.3; 16.1) 9 (3, 21)	**2651** **14.6 (20.4)** **(13.8; 15.4)** **9 (3, 21)**	0.014	2651	0
LOS/TBSA_2_		482 2.6 (4.7) CI (2.1; 3) 1.5 (0.8, 2.7)	2168 4.6 (17) CI (3.8; 5.3) 1.9 (0.8, 4)	**2650** **4.2 (15.5)** **CI (3.6; 4.8)** **1.8 (0.8, 3.6)**	0.002	2650	1
days_burn_to surgery_2_		198 20.2 (25.6) (16.6; 23.8) 16 (12, 21.8)	1449 18.3 (30.5) (16.8; 19.9) 14 (9, 20)	**1647** **18.6 (29.9)** **(17.1; 20)** **14 (10, 20)**	< 0.001	1647	1
In-hospital mortality_1_	Yes	0 (0%) CI (0; 0.8)	95 (4.3%) CI (3.5; 5.3)	**95 (3.5%)** **(2.9; 4.3)**	< 0.001	2651	0
30-day mortality_1_	Yes	0 (0%) CI (0; 0.8)	49 (2.4%) CI (1.8; 3.2)	**49 (2%)** **(1.5; 2.6)**	0.001^4^	2503	148
90-day mortality_1_	Yes	0 (0%) CI (0; 0.8)	49 (2.5%) CI (1.9; 3.3)	**49 (2%)** **(1.5; 2.7)**	0.001^4^	2416	235
Death (cause)_1_	MOF	0 (NaN%) CI (0; 100)	47 (49.5%) CI (39.1; 59.9)	**47 (49.5%)** **(39.1; 59.9)**		95	0
	CV	0 (NaN%) CI (0; 100)	15 (15.8%) CI (9.1; 24.7)	**15 (15.8%)** **(9.1; 24.7)**			
	ARDS	0 (NaN%) CI (0; 100)	13 (13.7%) CI (7.5; 22.3)	**13 (13.7%)** **(7.5; 22.3)**			
	SEPSIS	0 (NaN%) CI (0; 100)	9 (9.5%) CI (4.4; 17.2)	**9 (9.5%)** **(4.4; 17.2)**			
	Other	0 (NaN%) CI (0; 100)	11 (11.6%) CI (5.9; 19.8)	**11 (11.6%)** **(5.9; 19.8)**			

1: n(%)(Exact CI) *p* value: ^4^Chi^2^* p* value: ^3^Fisher's exact 2: N mean(sd) (mean 95% CI) median(IQR)* p* value: Mann–Whitney U.

*DVT* Deep vein thrombosis,* PE* pulmonary embolism,* CV* cardiovascular.

Significant values are in bold.

Mechanical ventilation was required in 3.9% of children and 9.4% of adults (*p* < 0.001). Median time on mechanical ventilation was significantly lower in children (6 days [IQR 3.15.5]) compared to adults (10 days [IQR 2.25]).

In adults, the most prevalent complications during hospital stay were sepsis (16.6%), AKI (7.9%), and cardiovascular complications (5.9%). Among children, the most frequent complication was sepsis (14.9%). The most frequent foci of infection in our sample were burns (50.7%), following by respiratory (35.2%), and urine (27.9%) infections.

Blood transfusion was required in 13.7% of patients; the rate of transfusion was significantly higher in adults vs. children (*p* < 0.001).

Overall mortality in the study period was 3.5%; the age-adjusted the mortality rate in children was 0% vs. 4.3% in adults. Table 6 describes the clinical differences between survivors and deceased.

**Table 6:** Description of mortality variables.

Variables		Survivors 2074 (95.62%)	Non-survivors (4.38%)	ALL	*p* value	n
Gender_1_	Men	1283 (61.9%) CI [59.7; 64]	60 (63.2%) CI [52.6; 72.8]	**1343 (61.9%)** **[59.8; 64]**	0.884^4^	2169
	Women	791 (38.1%) CI [36; 40.3]	35 (36.8%) CI [27.2; 47.4]	**826 (38.1%)** **[36; 40.2]**		
TBSA_2_		2074 7.2 (9.8) CI [6.8; 7.7] 4 [1.5, 9]	95 37.7 (28) CI [31.9; 43.4] 30 [13.5, 53.5]	**2169** **8.6 (12.8)** **CI [8; 9.1]** **4.5 [1.5, 10]**	< 0.001	2169
Delay_1_	> 24 h	687 (33.9%) CI [31.8; 36]	7 (7.4%) CI [3; 14.6]	**694 (32.7%)** **[30.7; 34.7]**	< 0.001	2124
	12 h a 24 h	93 (4.6%) CI [3.7; 5.6]	2 (2.1%) CI [0.3; 7.4]	**95 (4.5%)** **[3.6; 5.4]**		
	6 h a 12 h	176 (8.7%) CI [7.5; 10]	10 (10.5%) CI [5.2; 18.5]	**186 (8.8%)** **[7.6; 10]**		
	< 6 h	1073 (52.9%) CI [50.7; 55.1]	76 (80%) CI [70.5; 87.5]	**1149 (54.1%)** **[51.9; 56.2]**		
Smoker_1_	Yes	650 (31.3%) CI [29.3; 33.4]	34 (35.8%) CI [26.2; 46.3]	**684 (31.5%)** **[29.6; 33.5]**	0.424^4^	2169
Enolism_1_	Yes	161 (7.8%) CI [6.6; 9]	22 (23.2%) CI [15.1; 32.9]	**183 (8.4%)** **[7.3; 9.7]**	< 0.001	2169
Cocaine_1_	Yes	56 (2.7%) CI [2; 3.5]	3 (3.2%) CI [0.7; 9]	**59 (2.7%)** **[2.1; 3.5]**	0.742^3^	2169
Cannabis_1_	Yes	94 (4.5%) CI [3.7; 5.5]	1 (1.1%) CI [0; 5.7]	**95 (4.4%)** **[3.6; 5.3]**	0.125^3^	2169
Cardiovascular comorbidity_1_	Yes	596 (28.7%) CI [26.8; 30.7]	53 (57%) CI [46.3; 67.2]	**649 (29.9%)** **[28; 31.9]**	< 0.001	649
HTA_1_	Yes	545 (91.4%) CI [88.9; 93.6]	42 (79.2%) CI [65.9; 89.2]	**587 (90.4%)** **[87.9; 92.6]**	0.008^4^	649
Ischaemic heart disease_1_	Yes	73 (12.2%) CI [9.7; 15.2]	16 (30.2%) CI [18.3; 44.3]	**89 (13.7%)** **[11.2; 16.6]**	< 0.001	649
Heart failure_1_	Yes	38 (6.4%) CI [4.6; 8.6]	7 (13.2%) CI [5.5; 25.3]	**45 (6.9%)** **[5.1; 9.2]**	0.083^3^	649
Arrhythmias_1_	Yes	73 (12.2%) CI [9.7; 15.2]	18 (34%) CI [21.5; 48.3]	**91 (14%)** **[11.4; 16.9]**	< 0.001	649
Valvulopathy_1_	Yes	30 (5%) CI [3.4; 7.1]	7 (13.2%) CI [5.5; 25.3]	**37 (5.7%)** **[4; 7.8]**	0.024^3^	649
Vasculopathy_1_	Yes	36 (6%) CI [4.3; 8.3]	6 (11.3%) CI [4.3; 23]	**42 (6.5%)** **[4.7; 8.6]**	0.142^3^	649
Pulmonary comorbidity_1_	Yes	245 (11.8%) CI [10.5; 13.3]	15 (16.1%) CI [9.3; 25.2]	**260 (12%)** **[10.7; 13.5]**	0.28^4^	2162
Diabetes Melitus_1_	Yes	239 (11.5%) CI [10.2; 13]	21 (22.6%) CI [14.6; 32.4]	**260 (12%)** **[10.7; 13.4]**	0.002^4^	2167
Chronic kidney injury_1_	Yes	80 (3.9%) CI [3.1; 4.8]	5 (5.4%) CI [1.8; 12.1]	**85 (3.9%)** **[3.1; 4.8]**	0.41^3^	2167
Hepatopaty_1_	Yes	52 (2.5%) CI [1.9; 3.3]	5 (5.4%) CI [1.8; 12.1]	**57 (2.6%)** **[2; 3.4]**	0.095^3^	2167
Clotting disorders/OAC’s/APT_1_	Yes	178 (8.6%) CI [7.4; 9.9]	26 (28%) CI [19.1; 38.2]	**204 (9.4%)** **[8.2; 10.7]**	< 0.001	2167
Obesity_1_	Yes	595 (39.5%) CI [37; 42]	22 (41.5%) CI [28.1; 55.9]	**617 (39.5%)** **[37.1; 42]**	0.875^4^	1561
Chronic anaemia	Yes	75 (3.6%) CI [2.9; 4.5]	2 (2.2%) CI [0.3; 7.6]	**77 (3.6%)** **[2.8; 4.4]**	0.771^3^	2167
Dislipemia_1_	Yes	427 (20.6%) CI [18.9; 22.4]	29 (31.2%) CI [22; 41.6]	**456 (21.1%)** **[19.4; 22.8]**	0.021^4^	2165
Smoke inhalation_1_	No	1952 (95.5%) CI [94.5; 96.3]	46 (50%) CI [39.4; 60.6]	**1998 (93.5%)** **[92.4; 94.5]**	< 0.001	2137
	Yes	93 (4.5%) CI [3.7; 5.5]	46 (50%) CI [39.4; 60.6]	**139 (6.5%)** **[5.5; 7.6]**		
ABSI_2_		2071 4.8 (1.5) CI [4.8; 4.9] 5 [4, 6]	95 9.6 (2.6) CI [9.1; 10.1] 9 [8, 11.5]	**2166** **5 (1.9)** **CI [5; 5.1]** **5 [4, 6]**	< 0.001	2166
ARDS_1_	Yes	42 (2.1%) CI [1.5; 2.8]	43 (46.2%) CI [35.8; 56.9]	**85 (4%)** **[3.2; 4.9]**	< 0.001	2131
Age_1_	17 to 65	1624 (78.3%) CI [76.5; 80.1]	39 (41.1%) CI [31.1; 51.6]	**1663 (76.7%)** **[74.8; 78.4]**	< 0.001	2169
	65 to 80	301 (14.5%) CI [13; 16.1]	18 (18.9%) CI [11.6; 28.3]	**319 (14.7%)** **[13.2; 16.3]**		
	> 80	149 (7.2%) CI [6.1; 8.4]	38 (40%) CI [30.1; 50.6]	**187 (8.6%)** **[7.5; 9.9]**		
TBSA_1_	< 20	1908 (92%) CI [90.7; 93.1]	31 (32.6%) CI [23.4; 43]	**1939 (89.4%)** **[88; 90.7]**	< 0.001	2169
	> 20	166 (8%) CI [6.9; 9.3]	64 (67.4%) CI [57; 76.6]	**230 (10.6%)** **[9.3; 12]**		
Full thickness burn_1_	Yes	441 (21.3%) CI [19.5; 23.1]	66 (69.5%) CI [59.2; 78.5]	**507 (23.4%)** **[21.6; 25.2]**	< 0.001	2169
Mecanismo causal_1_	Others	1264 (60.9%) CI [58.8; 63.1]	25 (26.3%) CI [17.8; 36.4]	**1289 (59.4%)** **[57.3; 61.5]**	< 0.001	2169
	Flame	810 (39.1%) CI [36.9; 41.2]	70 (73.7%) CI [63.6; 82.2]	**880 (40.6%)** **[38.5; 42.7]**		
ABSI_cat	(0,3]	377 (18.2%) CI [16.6; 19.9]	1 (1.1%) CI [0; 5.7]	**378 (17.5%)** **[15.9; 19.1]**	< 0.001	2166
	(4,5]	1092 (52.7%) CI [50.6; 54.9]	0 (0%) CI [0; 3.8]	**1092 (50.4%)** **[48.3; 52.5]**		
	(6,7]	495 (23.9%) CI [22.1; 25.8]	20 (21.1%) CI [13.4; 30.6]	**515 (23.8%)** **[22; 25.6]**		
	(8,9]	85 (4.1%) CI [3.3; 5.1]	31 (32.6%) CI [23.4; 43]	**116 (5.4%)** **[4.4; 6.4]**		
	(10,11]	17 (0.8%) CI [0.5; 1.3]	19 (20%) CI [12.5; 29.5]	**36 (1.7%)** **[1.2; 2.3]**		
	(12,16]	5 (0.2%) CI [0.1; 0.6]	24 (25.3%) CI [16.9; 35.2]	**29 (1.3%)** **[0.9; 1.9]**		

1: by col n(%) [Exact CI] *p* value: ^4^Chi-squared *p* value: ^3^Fisher’s exac.

2: N mean(sd) [CI95% mean] median[IQR] *p* value: Mann–Whitney U.

Significant values are in bold.

**Figure 2:**
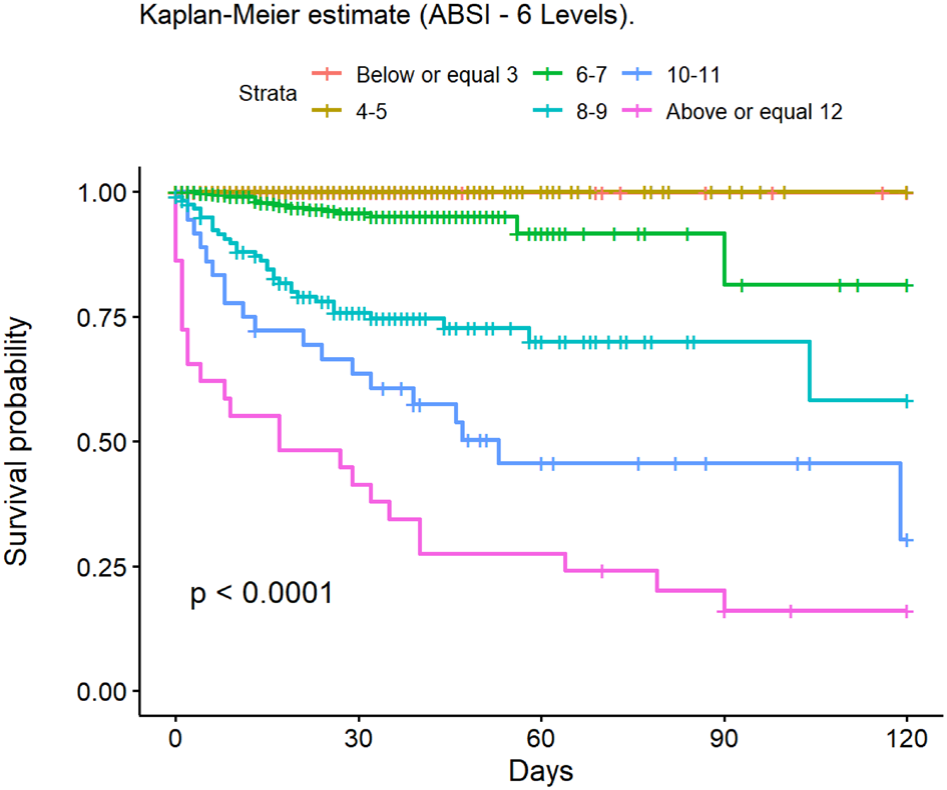
Kaplan–Meier survival curve for ABSI score.

**Figure 3:**
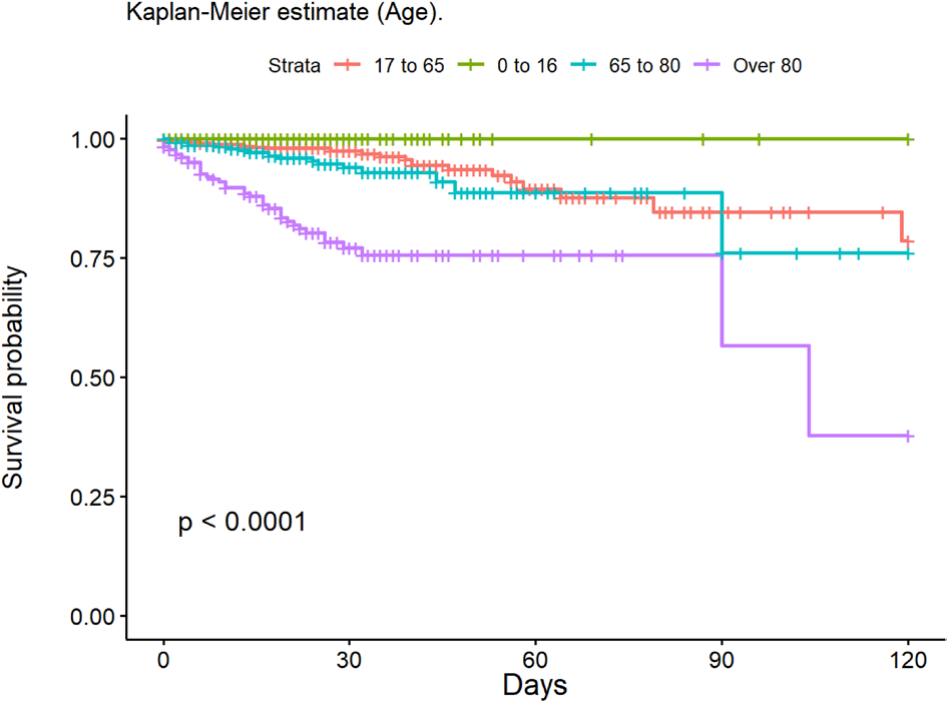
Kaplan–Meier survival curve for Age.

**Figure 4:**
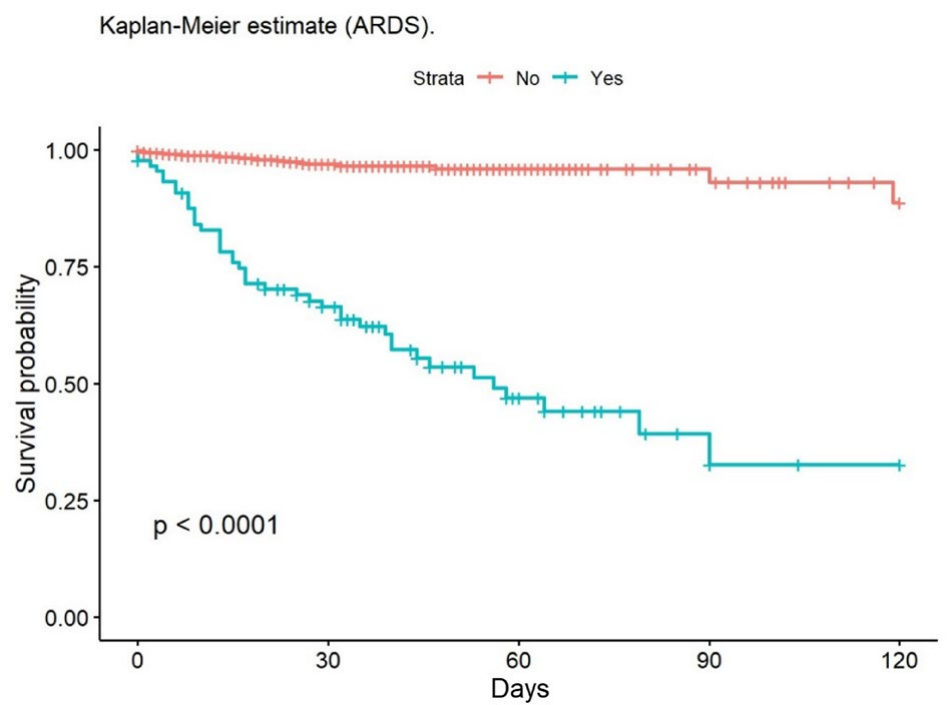
Kaplan–Meier survival curve for ARDS.

**Figure 5:**
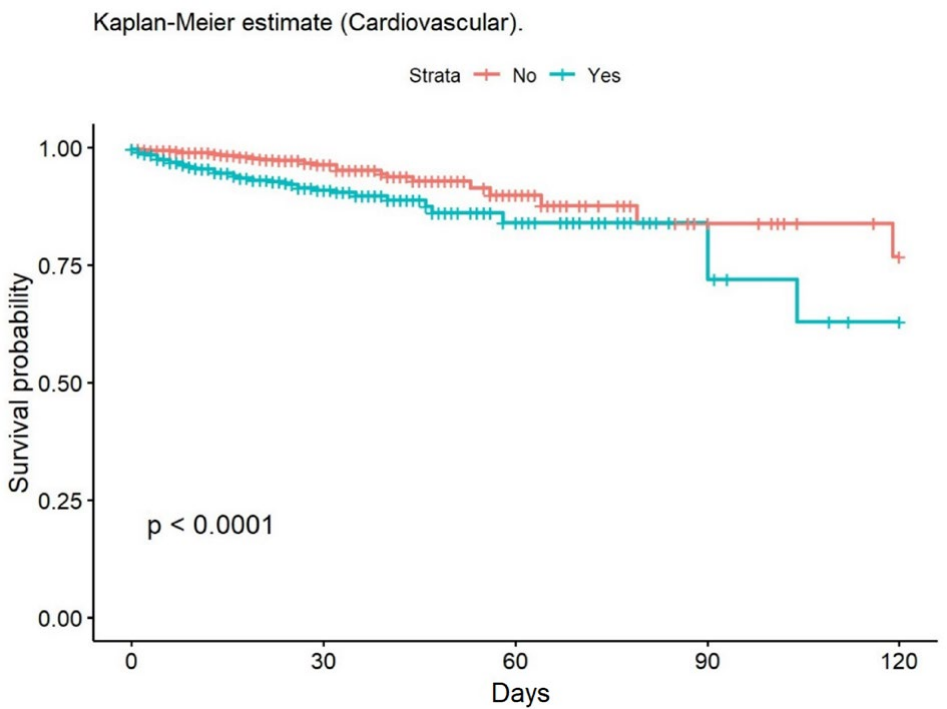
Kaplan–Meier survival curve for Cardiovascular comorbidity.

**Figure 6:**
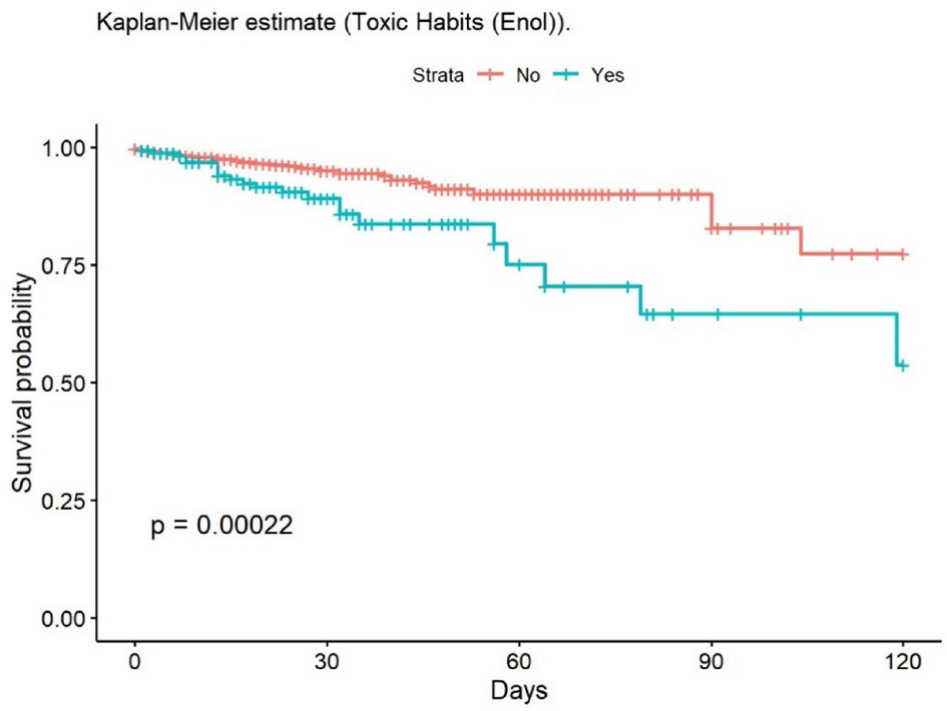
Kaplan–Meier survival curve for Enolism.

**Figure 7:**
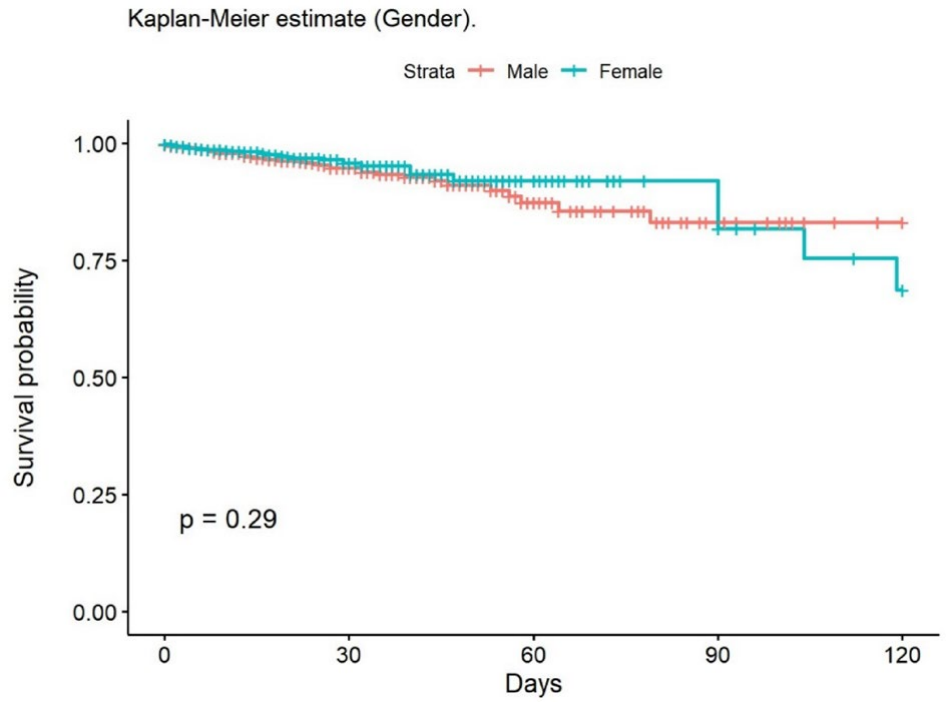
Kaplan–Meier survival curve for Gender.

**Figure 8:**
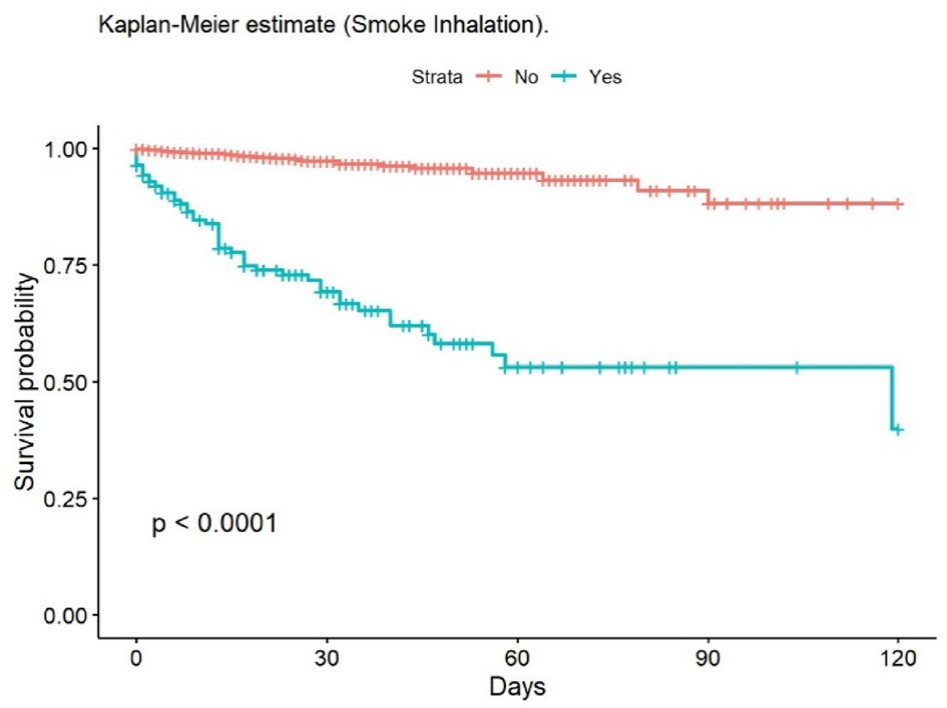
Kaplan–Meier survival curve for Smoke Inhalation.

**Figure 9:**
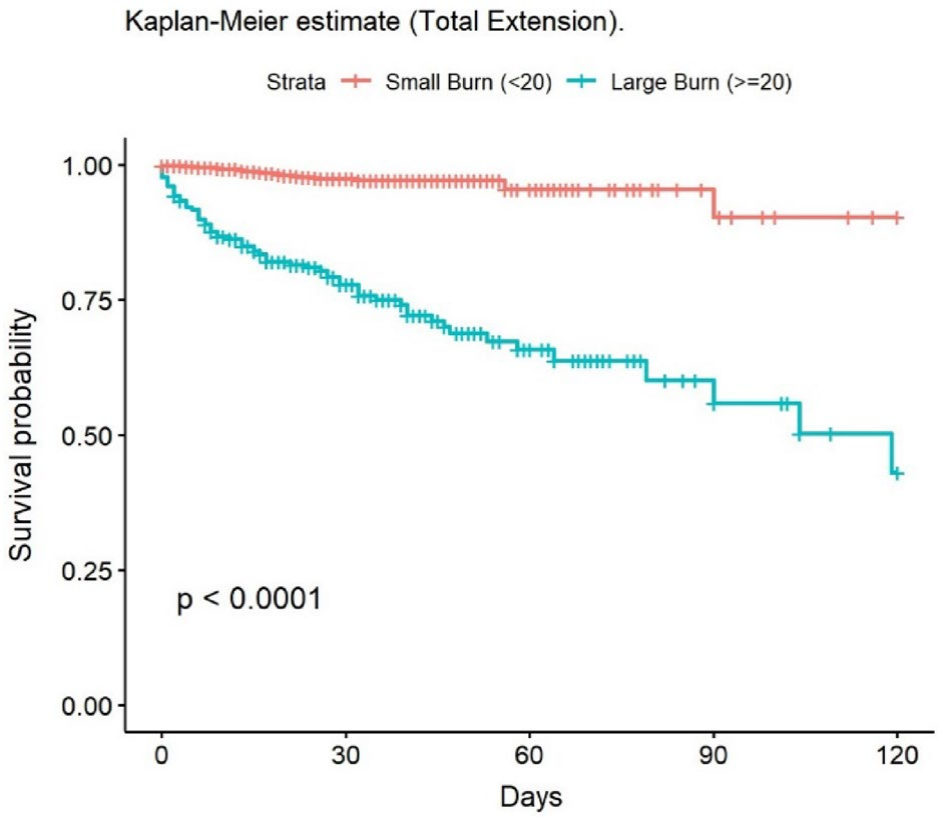
Kaplan–Meier survival curve for TBSA (%).

**Figure 10:**
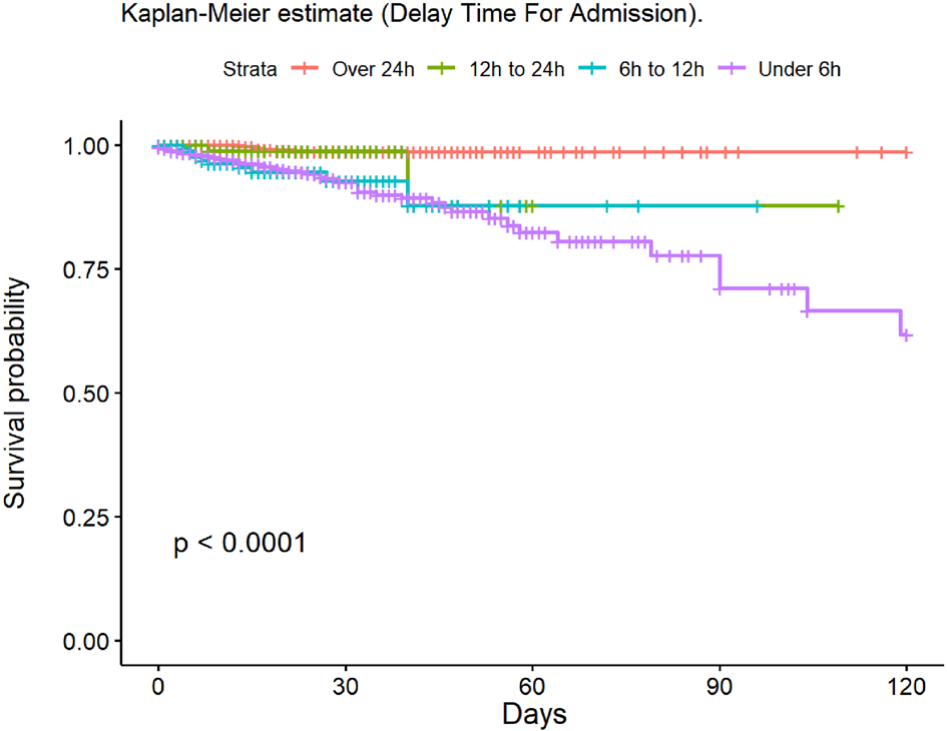
Kaplan–Meier survival curve for delay time for admission.

The most frequent cause of death was multiple organ failure (MOF) (49.5%); 9.5% of deaths were due to sepsis. Mortality at 30 and 90 days was 2% in the group of adults, while no children died during post-discharge follow-up. At 30 days, 148 patients had been lost to follow-up, and 235 at 90 days. The mean age of patients who died was 68.6 years. The mean LOS in our population was 14.6 (± 20.4) days; this was significantly lower in children (11.8 [± 14.8] days). The median LOS/TBSA ratio was 1.9 for adults and 1.5 for children. In the univariate analysis, the Kaplan–Meier survival curve was calculated (Figs. 2, 3, 4, 5, 6, 7, 8, 9 and 10) for each qualitative variable (Table 7).

**Table 7:** Cox Regression analysis of factors associated with mortality.

Variables	Hazard ratio	95% CI	*p* value
Sex	1.58	0.597–1.125	0.29
Age 0–16	inf	inf	inf
16–64	Ref	Ref	Ref
65–80	1.770	1.011–3.100	0.046
> 80	6.782	4.333–10.62	< 0.001
Mechanism: Flame	4.686	2.966–7.403	< 0.001
Mechanism: Other	Ref	Ref	Ref
ABSI < 7	Ref	Ref	Ref
ABSI ≥ 7	51.79	23.86–112.4	< 0.001
Delay > 6 h	Ref	Ref	Ref
Delay < 6 h	8.263	3.807–17.93	< 0.001
TBSA ≥ 20%	11.08	07:13–17.23	< 0.001
Smoke Inhalation	13.02	8.560–19.81	< 0.001
Enolism	2.428	1.495–3.944	< 0.001
Cardiovascular comorbidity	2.429	1.608–3.668	< 0.001
Clotting disorder	2.623	1.658–4.149	< 0.001
ARDS	14.70	9.609–22.49	< 0.001
TBSA% Full-thickness burn	6.820	3.689–12.61	< 0.001
TBSA (%)	1.047	1.040–1.054	< 0.001
ABSI	1.745	1.641–1.856	< 0.001

*CI* Confidence Interval.

In the clinical adjusted multivariate analysis (Table 8), the factors that increased mortality were ABSI ≥ 7 (HR 15.55 [95% CI 6.62–36.54]; *p* < 0.001), age over 80 years (HR 23.52 [95% CI 10.10–54.77] *p* < 0.001), smoke inhalation (HR 3.30 [95% CI 2.15–5.07]; *p* < 0.001), burned TBSA (1.02 [95% CI 1.02–1.03]; *p* < 0.001), cardiovascular comorbidity (HR 2.12 [95% CI 1.34–3.34]; *p* < 0.001), and full-thickness burn (1.69 [95% CI 1.06–2.68]; *p* = 0.027). Table 9 shows the multivariate analysis of factors associated with mortality (unadjusted). The observed mortality correlated with the ABSI-predicted mortality, with no statistically significant differences (Table 10).

**Table 8:** Multivariate analysis of factors related to mortality (adjusted for clinical factors).

Multivariate analysis	Hazard ratio	95% CI	*p* value
Age 65–80	4.85	2.28–10.31	** < 0.001**
Age 80	23.52	10.10–54.77	** < 0.001**
ABSI ≥ 7	15.55	6.62–36.54	** < 0.001**
Smoke inhalation	3.30	2.15–5.07	** < 0.001**
TBSA %	1.02	1.02–1.03	** < 0.001**
Cardiovascular comorbidity	2.12	1.34–3.34	**0.001**
Full thickness burn (YES/NO)	1.69	01:06–2.68	**0.027**

R^2^ Nagelkerke: 0.267 CI: Confidence Interval.

Significant values are in bold.

**Table 9:** Multivariate analysis of factors related to mortality (unadjusted).

	Hazard ratio	CI (lower)	CI (upper)	Pr( >|z|)
Age: 0 a 16	9.053e−08	0.000	Inf	0.997
Age: 65 a 80	4.651	1.704	12.70	0.003
Age: > 80	27.57	9.068	83.81	< 0.001
Flame	1.917	0.9183	4.00	0.083
ABSI ≥ 7	1.436	0.2972	6.939	0.652
Delay: 12 h a 24 h	1.589	0.1748	14.46	0.681
Delay: 6 h a 12 h	1.797	0.3706	8.713	0.467
Delay: < 6 h	2.574	0.8010	8.271	0.112
TBSA (%)	1.057	1.035	1.079	< 0.001
Enolism: Yes	2.036	0.8788	4.715	0.097
Cannabis: Yes	0.3402	0.09000	1.286	0.112
Chronic kidney injury: Yes	0.3851	0.1210	1.226	0.106
Hepatopaty: Yes	11.21	2.807	44.79	0.001
ARDS: Yes	0.7269	0.3736	1.414	0.348
Cardiovascular comorbidity	1.520	1.108	2.087	0.010
Smoke inhalation: Yes	2.750	1.408	5.369	0.003
TBSA Full thickness: 11 a 20%	1.828	0.8390	3.982	0.129
TBSA Full thickness: > 20%	1.205	0.4030	3.601	0.739

**Table 10:** Observed versus predicted mortality^67^.

ABSI	n observed	Observed mortality (%)	n predicted	Predicted mortality	*p* value_1_
2–3	1	0.26	4	< 1%	0.204
4–5	3	0.69	21	2%	0.218
6–7	19	12.7	51	10–20%	0.126
8–9	30	39.6	35	30–50%	0.414
10–11	19	72.4	22	60–80%	0.312
≥ 12	23	86.6	26	> 90%	0.178
Total	95		159		

1: Chi-squared.

### Economic factors

In our study, mean annual expenditure was €5,030,043.85, with a mean cost per patient of €15,179.31.

## Discussion

This is the first study to describe the epidemiological profile of children and adults presenting with burns in Catalonia. It is also the first to describe the overall in-hospital complications presented by patients admitted for burns, since these complications are usually analysed in a subgroup, such as major burn patients.

### Demographics

Burns were more frequent among men (61.6%)—a global trend due to the predominance of men working in metallurgy or agriculture. In our review, this incidence was somewhat lower than that reported in 1998 (64%), 2008 (65%), and 2018 (66%)^21,25,26^, and is consistent with the trend observed in other developed countries^6–8,15,27–30^. In Palacios^31^, however, 51% of burn patients were men.

The mean age in our study was 41.4 years (49.7 years in adults). This is similar to Palacios ^31^ and higher than the earlier study published by Barrett^21^, and reflects a global trend due to worldwide population ageing.

The proportion of burns in children (18.1%) is lower than in other developed countries^7,28,32,33^ and far lower than that reported in developing countries^11,34^. One of the reasons for this low incidence is the consistently low birth rate in Spain over the past few decades and compulsory school education. Interestingly, the age range with the highest incidence of burns is usually 0–4 years, in some cases accounting for up to 50% of all admissions^32,35–38^. In our study, the incidence in this age group was 68%. This percentage, though similar to that reported in other studies, is still very high, and suggests that the authorities should promote health education and implement preventive measures in this age group (including their parents). A relatively simply measure would be to introduce legislation to lower the maximum permitted temperature of household water, an approach that has proven effective in other countries^39,40^.

### Characteristic of burns

Scalding was the most frequent mechanism of injury (37.7%) in our review, whereas flame was the most prevalent cause of burns in the 1990s. After adjusting for age and sex, flame was the most prevalent mechanism in adult men, and scalding was the most prevalent mechanism in adult women and in children. These results are in line with studies published in other countries, such as Portugal^41^, Israel^29^, China^42–44^, Switzerland^9^, and Holland^7^. In Europe, flame was the most prevalent mechanism of injury, although scalding was more prevalent in children^27^.

The mean burned TBSA was 8.3%, significantly lower than previous studies, such as 16% in Barrett^21^ and 18% in Sánchez^25^. This reduction in burned TBSA may be due to a lower incidence of flame burns, which are usually severe, extensive, and accompanied by smoke inhalation^45^.

The proportion of patients with burns covering less than 10% of total body surface has increased from 60% in 1995 to 75% today. This is a global trend that has also been reported in other studies^7,16,43,46,47^, and could be due to the decline of flame as the main cause of burns in our setting. For the same reason, the number of patients with 20% of TBSA affected has decreased to 9.7%. According to figures from the ABA (American Burn Association), 22.5% of burn patients in 2013 presented major burns^48^. In children, 77% of patients presented burns affecting less than 10% TBSA, in line with other studies^28,35,36,41,49^.

### Comorbidities

Our study provides an overview of comorbidities in burn patients and their effect on mortality. Smoking is the most prevalent comorbidity (25.8%), while the rate of cardiovascular disease (24%) is similar to Barret et al. ^21^, and significantly higher than that reported by other authors, such as 8% in Knowlin^50^. The percentage of in-hospital cardiovascular complications was also higher in our series compared with Knowlin (5.9% vs. 3%), while cardiovascular comorbidity increased mortality at a rate similar to that observe by Knowlin^50^ (HR 2.12 [95% CI 1.34–3.34], *p* = 0.001).

Alcohol abuse was reported in 6.9% of patients in our study, a high rate compared to the average of 4.2% in the Spanish population in 2019^51^. These figures are consistent with those reported by Eiroa-Orosa, who showed that a higher proportion of burn patients had a history of substance abuse compared with the general population^52^.

In Spain, according to the ENPE study, 22% of the population is obese, and obesity is more prevalent in men aged over 65 years from low-income groups^53^ . In Catalonia, the percentage of obesity (16%) is lower than the Spanish average. In our series, 34.5% of adult patients were obese, a proportion that contrasts with most reports in the literature; however, according to the Center for Disease Control, 42.4% of the US population was obese in 2017^54^, while Jeschke reported a prevalence of 29%^55^. In our population, obesity was not associated with increased mortality.

### Treatment

Surgery was required in 64% of our patients, a percentage that is higher than other studies, such as Dokter^7^. The significant difference in the rate of surgery observed between adults and children is consistent with other studies, in which burns in children are usually less severe and respond well to conservative treatment, particularly high protein intake.

### Complications

None of the studies identified in our database search summarise the most common complications during hospital admission.

Several authors have described AKI as an independent risk factor for mortality^56–60^. This complication arose in 8% of patients in our study, whereas an incidence of 30% has been reported in other studies^60^ with an 80% mortality rate^56,61^. However, it is important to bear in mind that nearly all these studies were performed in critically ill patients^17,62^, so the findings cannot be extrapolated to the general population. Burn patients are at increased risk of suffering potentially fatal infection of any cause in the first 5 years after the burn^63^, and sepsis increases LOS and ICULOS^64^. In our study, 16.3% of patients were diagnosed with sepsis, somewhat higher than the figures published in Belgium^65^.

### Outcomes

Mortality remained at 3.5% (0% in children under 16 years of age and 4.3% in adults). This rate is somewhat higher than that reported in most developed European countries^6,10,15,65,66^.

We compared observed vs. predicted mortality based on the ABSI to determine the accuracy of this score in our population^67^. The ABSI correlated with observed mortality, except in mild injury patients (ABSI < 6) and very severe injury patients (ABSI ≥ 12), in which mortality was lower than predicted.

A factor that has changed substantially over the years is cause of death. In Barrett et al., the main causes were acute respiratory distress syndrome (ARDS) (34%), MOF (26.8%) and sepsis (13.2%). The decrease in ARDS (13.7%) is striking, and may be due to improvements in critical care, a decrease in flame burns, and advances in mechanical ventilation. The incidence of sepsis-related deaths has also declined, and it is now the cause of death in 9.5% of patients, 50% lower than the figure reported by Barret et al.^21^ and in line with the findings of a Belgian study^65^. The decrease in mortality due to sepsis is a promising development, and may be due to the implementation of strict care protocols in critically ill patients^68^, together with improvements in the use of antibiotics and in the management of septic shock.

In our study, the main cause of death was MOF (49.5%). According to the American Burn Association, MOF is the cause in 27.5% of fatalities^48,69^ . In other studies, this percentage increases to 40%.^17,70^.

The main predictors of in-hospital mortality identified in this study are age over 80 years, ABSI ≥ 7, smoke inhalation, cardiovascular comorbidity, full-thickness burn, and burned TBSA. These factors have also been described as predictors elsewhere^8,27,33,43,46,50,71,72^. Interestingly, there is scant evidence elsewhere of the association between cardiovascular comorbidity and increased mortality; however, in our series, this comorbidity increased the probability of dying by 112%. We, like some other authors, did not find gender to be associated with increased mortality^8,73,74^, although this contrasts with the findings of other studies^67^.

The number of admissions for burns has decreased dramatically from 6.6/10^5^ population/year to 3.68/10^5^ population/year This is a global trend^1,2,15,33,75^, despite significantly higher rates of admission reported in some of our European neighbours, such as 18.9/10^5^^41^ in Portugal, and 36.9/10^5^ in Romania.^11^ Both LOS and LOS/TBSA in Catalonia are above the average in developed countries^2,4,6,11,13,71^, possibly due to 2 factors: first, the average waiting time from burn event to surgery is 18 days, far higher than the average 14.7 days in the Netherlands^7^; second, home care in our region is underdeveloped, a situation that places an additional burden on in-hospital care.

In total, 14% of adults were admitted for burns occurring in the workplace, similar to the percentage reported by Barrett, and midway between the 5.9% reported by Palacios^31^ and 20.9% by Sánchez^25^. Other countries that have published statistics relating to burns in the workplace include Germany, with an incidence that ranges from 18%-33.7%^12,76^, Switzerland with 31%^9^, the USA with 18%^77^, China with 78%^72^, Australia with 17% ^74,78^, and Austria with 14.9%^8^.

### Economic factors

Care costs are currently one of the main problems in the healthcare sector. The economic downturn followed by the SARS-CoV-2 pandemic has brought public health systems in Spain to the brink of collapse, and it is now more important than ever to optimise resource management. As mentioned above, mean annual expenditure was €5,030,043.85, with a mean cost per patient of €15,179.31; these cost estimates were not TBSA-weighted. In Portugal, meanwhile, the cost per patient is €8,030^41^, in Holland it is €26,540^30^ and in Finland, €25,000^3^.

This study has several limitations.

As our study was performed in a single hospital, our findings cannot be assumed to reflect the situation in other regions; however, this single-centre design ensures that both the study population and the treatment of burns is homogeneous.

Selection bias cannot be ruled out, since patients with non-severe burns are not initially treated at or transferred to the HUVH, so we were unable to include these data in our analysis. Another potential source of selection bias stems from our retrospective design, insofar as some patients were lost because doctors did not always strictly adhere to the 90-day follow-up schedule.

In the study of comorbidities, patients were not assessed using the gold-standard Charlson index, and the presence of psychiatric disorders was not recorded.

## Conclusions

This study shows that changes have occurred in the pattern of burn injuries, their extent, and their severity, and burns are now predominantly less extensive and deep in our setting. The pattern of clinical presentation differed been children and adults. In our series, children ages between 0 and 4 years of age account for 68% of all children admitted for burns. This means that they are still most important risk group and should be the target of preventive measures and health education campaigns. The factors associated with a higher risk of mortality were age, %TBSA, full-thickness burns, smoke inhalation, and cardiovascular comorbidity. Unlike other studies, in our series female sex was not a risk factor for mortality. Prospective, multicentre studies are needed to obtain a more accurate picture of the situation of burn patients in Spain.

### Supplementary Information

Supplementary Information.

## Data Availability

The data collected in this study is available upon reasonable request. Please, contact with Luis Abarca Vilchez; labarcavilchez@gmail.com.

## Abbreviations

TBSA: Total body surface area
LOS: Length of stay
AKI: Acute kidney injury
ABSI: Abbreviated Burn Severity Index
MOF: Multiple Organ Failure
HUVH: Hospital Universitario Vall d’ Hebrón

## References

[1] James SL, Lucchesi LR, Bisignano C, Castle CD, Dingels ZV, Fox JT (2019). Epidemiology of injuries from fire, heat and hot substances: Global, regional and national morbidity and mortality estimates from the Global Burden of Disease 2017 study. Inj. Prev..

[2] Smolle C, Cambiaso-Daniel J, Forbes AA, Wurzer P, Hundeshagen G, Branski LK (2017). Recent trends in burn epidemiology worldwide: A systematic review. Burns.

[3] Haikonen K, Lillsunde PM, Vuola J (2014). Inpatient costs of fire-related injuries in Finland. Burns.

[4] Kruger E, Kowal S, Pinar Bilir S, Han E, Foster K (2020). Relationship between patient characteristics and number of procedures as well as length of stay for patients surviving severe burn injuries: Analysis of the american burn association national burn repository. J. Burn Care Res..

[5] Guilabert P, Usúa G, Martín N, Abarca L, Barret JP, Colomina MJ (2016). Fluid resuscitation management in patients with burns: Update. Br. J. Anaesth..

[6] Åkerlund E, Huss FRM, Sjöberg F (2007). Burns in Sweden: An analysis of 24 538 cases during the period 1987–2004. Burns.

[7] Dokter J, Vloemans AF, Beerthuizen GIJM, van der Vlies CH, Boxma H, Breederveld R (2014). Epidemiology and trends in severe burns in the Netherlands. Burns.

[8] Ederer IA, Hacker S, Sternat N, Waldmann A, Salameh O, Radtke C (2019). Gender has no influence on mortality after burn injuries: A 20-year single center study with 839 patients. Burns.

[9] Müller M, Moser EM, Pfortmueller CA, Olariu R, Lehmann B, Exadaktylos AK (2016). Aetiology of adult burns treated from 2000 to 2012 in a Swiss University Hospital. Burns.

[10] Onarheim H, Jensen SA, Rosenberg BE, Guttormsen AB (2009). The epidemiology of patients with burn injuries admitted to Norwegian hospitals in 2007. Burns.

[11] Pieptu V, Moscalu R, Mihai A, Moscalu M, Pieptu D, Azoicăi D (2021). Epidemiology of hospitalized burns in Romania: A 10-year study on 92,333 patients. Burns.

[12] Schiefer JL, Perbix W, Grigutsch D, Zinser M, Demir E, Fuchs PC (2016). Etiology, incidence and gender-specific patterns of severe burns in a German Burn Center—Insights of 25 years. Burns.

[13] Tanttula K, Haikonen K, Vuola J (2018). Hospitalized burns in Finland: 36 305 cases from 1980–2010. Burns.

[14] Theodorou P, Xu W, Weinand C, Perbix W, Maegele M, Lefering R (2013). Incidence and treatment of burns: A twenty-year experience from a single center in Germany. Burns.

[15] van Yperen DT, van Lieshout EMM, Verhofstad MHJ, van der Vlies CH (2021). Epidemiology of burn patients admitted in the Netherlands: A nationwide registry study investigating incidence rates and hospital admission from 2014 to 2018. Eur. J. Trauma Emerg. Surg..

[16] Zayakova Y, Vajarov I, Stanev A, Nenkova N, Hristov H (2014). Epidemiological analysis of burn patients in East Bulgaria. Burns.

[17] Jeschke MG, Pinto R, Kraft R, Nathens AB, Finnerty CC, Gamelli RL (2015). Morbidity and survival probability in burn patients in modern burn care. Crit. Care Med..

[18] Knowlin L, Stanford L, Moore D, Cairns B, Charles A (2016). The measured effect magnitude of co-morbidities on burn injury mortality. Burns.

[19] Low ZK, Ng WY, Fook-Chong S, Tan BK, Chong SJ, Hwee J (2017). Comparison of clinical outcomes in diabetic and non-diabetic burns patients in a national burns referral centre in southeast Asia: A 3-year retrospective review. Burns.

[20] Sayampanathan AA (2016). Systematic review and meta-analysis of complications and outcomes of obese patients with burns. Burns.

[21] Barret JP, Gomez P, Solano I, Gonzalez-Dorrego M, Crisol FJ (1999). Epidemiology and mortality of adult burns in Catalonia 1999. Burns.

[22] Greenhalgh DG, Saffle JR, Holmes JH, Gamelli RL, Palmieri TL, Horton JW (2007). American burn association consensus conference to define sepsis and infection in burns. J. Burn Care Res..

[23] Putra ON, Saputro ID, Diana D (2021). Rifle criteria for acute kidney injury in burn patients: prevalence and risk factors. Ann. Burns Fire Disasters.

[24] Cartotto R, Li Z, Hanna S, Spano S, Wood D, Chung K (2016). The Acute Respiratory Distress Syndrome (ARDS) in mechanically ventilated burn patients: An analysis of risk factors, clinical features, and outcomes using the Berlin ARDS definition. Burns.

[25] Sanchez JLA, Bastida JL, Martínez MM, Moreno JMM, Chamorro JJ (2008). Socio-economic cost and health-related quality of life of burn victims in Spain. Burns.

[26] Sousa D, Ceniceros A, Galeiras R, Pértega-Díaz S, Gutiérrez-Urbón JM, Rodríguez-Mayo M (2018). Microbiology in burns patients with blood stream infections: Trends over time and during the course of hospitalization. Infect. Dis..

[27] Brusselaers N, Monstrey S, Vogelaers D, Hoste E, Blot S (2010). Severe burn injury in Europe: A systematic review of the incidence, etiology, morbidity, and mortality. Crit. Care.

[28] Duke J, Wood F, Semmens J, Edgar DW, Spilsbury K, Hendrie D (2011). A study of burn hospitalizations for children younger than 5 years of age: 1983–2008. Pediatrics.

[29] Harats M, Peleg K, Givon A, Kornhaber R, Goder M, Jaeger M (2016). Burns in Israel, comparative study: Demographic, etiologic and clinical trends 1997–2003 versus 2004–2010. Burns.

[30] Hop MJ, Wijnen BFM, Nieuwenhuis MK, Dokter J, Middelkoop E, Polinder S (2016). Economic burden of burn injuries in the Netherlands: A 3 months follow-up study. Injury.

[31] Palacios García P, Pacheco Compaña FJ, Rodríguez Pérez E, Bugallo Sanz JI, Fernández-Quinto A, Avellaneda-Oviedo EM (2020). Trends in burn injuries in Galicia (Spain): An epidemiological study. Int. Wound J..

[32] Duke JM, Rea S, Boyd JH, Randall SM, Wood FM (2015). Mortality after burn injury in children: A 33-year population-based study. Pediatrics.

[33] Zavlin D, Chegireddy V, Boukovalas S, Nia AM, Branski LK, Friedman JD (2018). Multi-institutional analysis of independent predictors for burn mortality in the United States. Burns Trauma.

[34] Li H, Wang S, Tan J, Zhou J, Wu J, Luo G (2017). Epidemiology of pediatric burns in southwest China from 2011 to 2015. Burns.

[35] Armstrong M, Wheeler KK, Shi J, Thakkar RK, Fabia RB, Groner JI (2021). Epidemiology and trend of US pediatric burn hospitalizations, 2003–2016. Burns.

[36] Johnson EL, Maguire S, Hollén LI, Nuttall D, Rea D, Kemp AM (2017). Agents, mechanisms and clinical features of non-scald burns in children: A prospective UK study. Burns.

[37] Sanyaolu L, Javed MU, Eales M, Hemington-Gorse S (2017). A 10 year epidemiological study of paediatric burns at the Welsh Centre for burns and plastic surgery. Burns.

[38] Wang S, Li D, Shen C, Chai J, Zhu H, Lin Y (2016). Epidemiology of burns in pediatric patients of Beijing City. BMC Pediatr..

[39] Kendrick D, Young B, Mason-Jones AJ, Ilyas N, Achana FA, Cooper NJ (2012). Home safety education and provision of safety equipment for injury prevention. Cochrane Database of Syst. Rev..

[40] Prokopenko M, Reed AJM, Chicco M, Issa F (2022). Preventable burns from domestic tap water. Eur. Burn J..

[41] Santos JV, Viana J, Oliveira A, Ramalho A, Sousa-Teixeira J, Duke J (2019). Hospitalisations with burns in children younger than five years in Portugal, 2011–2015. Burns.

[42] Chen SH, Chen YC, Chen TJ, Ma H (2014). Epidemiology of burns in Taiwan: A nationwide report including inpatients and outpatients. Burns.

[43] Cheng W, Wang S, Shen C, Zhao D, Li D, Shang Y (2018). Epidemiology of hospitalized burn patients in China: A systematic review. Burns Open.

[44] Fan X, Ma B, Zeng D, Fang X, Li H, Xiao S (2017). Burns in a major burns center in East China from 2005 to 2014: Incidence and outcome. Burns.

[45] Wasiak J, Spinks A, Ashby K, Clapperton A, Cleland H, Gabbe B (2009). The epidemiology of burn injuries in an Australian setting, 2000–2006. Burns.

[46] Chen L, He X, Xian J, Liao J, Chen X, Luo Y (2021). Development of a framework for managing severe burns through a 17-year retrospective analysis of burn epidemiology and outcomes. Sci. Rep..

[47] Cleland H, Fernando DT, Gabbe BJ (2021). Trends in Victorian burn injuries 2008–2017. Burns.

[48] Ogura A, Tsurumi A, Que YA, Almpani M, Zheng H, Tompkins RG (2019). Associations between clinical characteristics and the development of multiple organ failure after severe burns in adult patients. Burns.

[49] Brink C, Isaacs Q, Scriba MF, Nathire MEH, Rode H, Martinez R (2019). Infant burns: A single institution retrospective review. Burns.

[50] Knowlin L, Reid T, Williams F, Cairns B, Charles A (2017). Burn mortality in patients with preexisting cardiovascular disease. Burns.

[51] Observatorio Español de las Drogas y las Adicciones. Informe 2021. Alcohol, tabaco y drogas ilegales en España. Madrid: Ministerio de Sanidad. Delegación del Gobierno para el Plan Nacional sobre Drogas. 243 p. (2021).

[52] Eiroa-Orosa FJ, Giannoni-Pastor A, Fidel-Kinori SG, Argüello JM (2016). Substance use and misuse in burn patients: Testing the classical hypotheses of the interaction between post-traumatic symptomatology and substance use. J. Addict. Dis..

[53] Pérez-Rodrigo C, Hervás Bárbara G, Gianzo Citores M, Aranceta-Bartrina J (2021). Prevalence of obesity and associated cardiovascular risk factors in the Spanish population: The ENPE study. Revista Española de Cardiología (English Edition).

[54] Hales CM, Carroll MD, Fryar CD, Ogden CL. Prevalence of Obesity and Severe Obesity Among Adults: United States, 2017-2018 Key findings Data from the National Health and Nutrition Examination Survey. 2017.

[55] Mild Obesity Is Protective After Severe Burn Injury n.d. 10.1097/SLA.0b013e3182984d19 PMC3963145 23877367

[56] Clark A, Neyra JA, Madni T, Imran J, Phelan H, Arnoldo B (2017). Acute kidney injury after burn. Burns.

[57] Demsey D, Mordhorst A, Griesdale DEG, Papp A (2019). Improved outcomes of renal injury following burn trauma. Burns.

[58] Folkestad T, Brurberg KG, Nordhuus KM, Tveiten CK, Guttormsen AB, Os I (2020). Acute kidney injury in burn patients admitted to the intensive care unit: A systematic review and meta-analysis. Crit. Care.

[59] Witkowski W, Kawecki M, Surowiecka-Pastewka A, Klimm W, Szamotulska K, Niemczyk S (2016). Early and late acute kidney injury in severely burned patients. Med. Sci. Monit..

[60] Wu G, Xiao Y, Wang C, Hong X, Sun Y, Ma B (2017). Risk factors for acute kidney injury in patients with burn injury: A meta-analysis and systematic review. J. Burn Care Res..

[61] Kuo G, Yang SY, Chuang SS, Fan PC, Chang CH, Hsiao YC (2016). Using acute kidney injury severity and scoring systems to predict outcome in patients with burn injury. J. Formos. Med. Assoc..

[62] Tan Chor Lip H, Tan JH, Thomas M, Imran FH, Azmah Tuan Mat TN (2019). Survival analysis and mortality predictors of hospitalized severe burn victims in a Malaysian burns intensive care unit. Burns Trauma.

[63] Duke JM, Randall SM, Wood FM, Boyd JH, Fear MW (2017). Burns and long-term infectious disease morbidity: A population-based study. Burns.

[64] Raz-Pasteur A, Hussein K, Finkelstein R, Ullmann Y, Egozi D (2013). Blood stream infections (BSI) in severe burn patients-early and late BSI: A 9-year study. Burns.

[65] Costescu Strachinaru DI, Gallez JL, François PM, Baekelandt D, Paridaens MS, Pirnay JP (2021). Epidemiology and etiology of blood stream infections in a Belgian burn wound center. Acta Clin. Belg. Int. J. Clin. Lab. Med..

[66] Brandão C (2021). The role of comorbidities on outcome prediction in acute burn patients place des comorbidités dans les critères pronostiques des patients brûlés. Ann. Burns Fire Disasters.

[67] Forster NA, Zingg M, Haile SR, Künzi W, Giovanoli P, Guggenheim M (2011). 30 years later—Does the ABSI need revision?. Burns.

[68] Palomar M, Álvarez-Lerma F, Riera A, Díaz MT, Torres F, Agra Y (2013). Impact of a national multimodal intervention to prevent catheter-related bloodstream infection in the ICU: The Spanish experience. Crit. Care Med..

[69] Miller SF, Bessey PQ, Schurr MJ, Browning SM, Jeng JC, Caruso DM (2006). National burn repository 2005: A ten-year review. J. Burn Care Res..

[70] Kallinen O, Maisniemi K, Böhling T, Tukiainen E, Koljonen V (2012). Multiple organ failure as a cause of death in patients with severe burns. J. Burn Care Res..

[71] Nickel KJ, Omeis T, Papp A (2020). Demographics and clinical outcomes of adult burn patients admitted to a single provincial burn centre: A 40-year review. Burns.

[72] Wang T, Nie C, Zhang H, Zeng X, Yu H, Wei Z (2018). Epidemiological characteristics and factors affecting length of hospital stay for children and adults with burns in Zunyi, China: A retrospective study. PeerJ.

[73] Blom L, Klingberg A, Laflamme L, Wallis L, Hasselberg M (2016). Gender differences in burns: A study from emergency centres in the Western Cape, South Africa. Burns.

[74] Toppi J, Cleland H, Gabbe B (2019). Severe burns in Australian and New Zealand adults: Epidemiology and burn centre care. Burns.

[75] Mehta K, Arega H, Smith NL, Li K, Gause E, Lee J (2021). Gender-based disparities in burn injuries, care and outcomes: A World Health Organization (WHO) Global Burn Registry cohort study. Am. J. Surg..

[76] Obed D, Schroeter A, Gruber L, Bucher F, Salim M, Bingoel AS (2022). Epidemiology and outcome analysis of 1359 intensive care burn patients: A 13-year retrospective study in a major burn center in Germany. Burns.

[77] Nurczyk K, Chrisco LP, di Corpo M, Nizamani R, Sljivic S, Calvert CT (2020). Work-Related burn injuries in a tertiary care burn center, 2013 to 2018. J. Burn Care Res..

[78] McInnes JA, Cleland H, Tracy LM, Darton A, Wood FM, Perrett T (2019). Epidemiology of work-related burn injuries presenting to burn centres in Australia and New Zealand. Burns.

