# Supplementary material for: Epidemiology and mortality in patients hospitalized for burns in Catalonia, Spain
Source: Sci Rep. 2023 Sep 1;13:14364. doi: 10.1038/s41598-023-40198-2 (PMC10474035; doi:10.1038/s41598-023-40198-2)
Supplement: Supplementary file 1 — Supplementary Information. [file 41598_2023_40198_MOESM1_ESM.docx]

**Appendix 1: Definition of variables**

- Age: based on date of birth.

- Sex: male or female.

-TBSA: Total burned body surface area on admission, as described in the Lund and Browder chart.

- Anatomical location of the burn on admission, according to the chart.

- Burn degree, according to the chart.

- Mechanism of injury.

- Work-related burn.

- Toxic habits. Smoking, chronic alcoholism, cannabis, cocaine, or other substances classified as drugs.

- Cardiovascular: this category includes hypertension, ischaemic heart disease, arrhythmia, peripheral vascular disease, dilated or hypertrophic cardiomyopathy, and moderate-to-severe valvular heart disease.

- Respiratory: including history of COPD, bronchial asthma, pulmonary fibrosis or previously diagnosed interstitial diseases.

- Diabetes Mellitus 1 or 2.

- Chronic kidney failure. Patients with a glomerular filtration rate of less than 60 ml/min/m^2^ (measured by CKD-EPI) for at least 3 months in laboratory tests prior to admission, or documented structural or functional kidney injury. Staging according to KDIGO (Kidney Disease Improving Global Outcomes) criteria.

- Liver disease: patients diagnosed with liver cirrhosis.

- Clotting disorder: patients diagnosed with congenital or acquired clotting disorders, and patients undergoing antiplatelet or anticoagulant treatment.

- Chronic anaemia.

- Neuromuscular disorders: including epilepsy, ALS, MS, and myopathies.

- Active neoplasm at the time of the burn.

- Obesity: diagnosed in primary care or BMI > 30 documented in preoperative records.

- ABSI: Abbreviated Burn Severity Index.

- Smoke inhalation: if smoke inhalation was suspected in the admission report or diagnosed by fibreoptic bronchoscopy.

- ICU admission.

- LOS: length of hospital stay

- ICULOS: length of stay in the ICU.

- Surgical intervention: The surgery involves debriding the burns and placing an autograft or allograft depending on the patient's clinical condition.

- Re-intervention: need for re-intervention (involved debriding a previously non-debrided area due to its large size or because the grafted area had failed) and the number of reinterventions required.

- Compartment syndrome in extremities, chest, or abdominal compartment syndrome.

- Cardiovascular complications: arrhythmia, heart failure, or acute myocardial infarction.

- Adult respiratory distress syndrome (ARDS) diagnosed according to the Berlin ARDS criteria.

- Tracheostomy: if performed, time (in days) from intubation to tracheostomy.

- Days on mechanical ventilation.

- Acute kidney injury: RIFLE clinical criteria were followed.

- Stroke: stroke or no stroke during hospital stay.

- Sepsis: diagnosis was made following ABA (American Burn Association) recommendations. The origin of the culture was recorded.

- Mortality: date of death, main diagnosis, and the time (in days) from the date of the burn were recorded.

- MOF (Multiple Organs Failure) was defined as the failure of two or more of the following organs or systems: cardiovascular, respiratory, neurological, renal, hematological, gastrointestinal, hepatic, and neurological.

- Mortality at 30 and 90 days: this variable was collected from the plastic surgeon’s follow-up records. Patients with minor burns are not always followed up by the treating doctor, so primary care patient records were reviewed to ascertain whether the patients had been seen within 30 and 90 days after discharge in order to determine whether they were still surviving.
